# The Specific and Total CO_2_ Emission Activity of Wood-Decaying Fungi and Their Response to Increases in Temperature

**DOI:** 10.3390/jof10070448

**Published:** 2024-06-26

**Authors:** Victor A. Mukhin, Daria K. Diyarova, Elena V. Zhuykova

**Affiliations:** Institute of Plant and Animal Ecology, Ural Branch of the Russian Academy of Sciences, Ekaterinburg 620144, Russia; dasha_d@ipae.uran.ru (D.K.D.); zhuykova_ev@ipae.uran.ru (E.V.Z.)

**Keywords:** fungi, Basidiomycota, respiration, CO_2_, emission, temperature, climate

## Abstract

The CO_2_ emission activity of xylotrophic fungi responding to an increase in temperature in the range of 10–30 °C with pure dikaryotic cultures of *Fomes fomentarius* s. str., *F. inzengae*, *Fomitopsis betulina*, *F. pinicola*, and *Phellinus igniarius* was analyzed. Emission activity was assessed by the difference in CO_2_ concentration in 0.5 L exposure chambers with Petri dishes with mycelium growing on agar at the beginning of exposure and an hour later using a Gasmet DX-4030 FTIR spectrometer (Gasmet Technologies Oy, Finland), error measurements ±50 ppm. Specific (μg CO_2_/cm^2^/h) and total (μg CO_2_/h) emission activity and its relationship with temperature and size (area) of the mycelium were assessed. It is shown that in the range of 10–30 °C, the specific and total CO_2_ emission activity of the mycelium is closely and positively related to temperature. Specific emission, which is an indicator of the respiratory activity of the mycelium, does not depend on its size; its only driver is temperature, the relationship with which is linear: an increase in temperature by 10 °C causes an increase in the specific emission activity of the mycelium by 1.7 times. The total CO_2_ emission activity, which is an indicator of the total amount of CO_2_ emitted, is directly proportional to the specific emission activity and the size of the mycelium. In the range of 10–30 °C, an increase in temperature causes an almost equal increase in both the specific emission activity of the mycelium (Q_10_ 1.7) and its growth (Q_10_ 1.5) and causes an exponential increase in the total emission of CO_2_. This must be taken into account when predicting CO_2_ emissions from woody debris under climate change, as it could potentially contribute to accelerating climate change.

## 1. Introduction

Climate change, its causes, and environmental consequences are some of the most discussed problems in ecology, as well as in works on the ecology of woody debris and its decomposition [1,2,3,4]. This is far from accidental, since woody debris is a globally significant natural reservoir of carbon; in Russian forests alone, its reserves amount to about 5.5 Gt. This huge mass of woody debris is at various stages of its biological decomposition—a large-scale process specific to forest ecosystems, during which annually about 214 Mt C-CO_2_ is emitted into the atmosphere from Russian forests. This makes debris the second largest natural source of carbon dioxide after soil [1,5].

The main role in the decomposition of debris in forests of temperate latitudes is played by xylotrophic fungi—Basidiomycota and Agaricomycetes [6,7,8,9]. This is, perhaps, the only group of organisms in the modern biosphere that has a unique set of interconnected ecological and physiological adaptations to the woody habitat and is capable of decomposing the lignocellulosic complex of wood [10]. They determine the main parameters of CO_2_ emission activity of woody debris, and this makes them one of the globally significant regulators of the gas composition of the atmosphere [11], which is a factor of climate stability and change [4].

One of the key questions arising from climate change relates to future carbon dynamics, which largely depend on the temperature sensitivity of decomposition processes. They play an important role in the global carbon cycle and, through feedback, can potentially influence climate change [4,12,13]. All currently available data clearly indicate a close connection between the CO_2_ emission activity of xylotrophic fungi, wood debris, and temperature [2,3,4,14,15,16,17]. In particular, in one of our recent works [18], we showed that an increase in temperature from 20 to 30 °C has a nonadditive, possibly synergistic effect on the CO_2_ emission activity of xylotrophic fungi, causing its exponential growth.

Considering the role of xylotrophic fungi as factors of stability and climate change, this phenomenon undoubtedly requires most careful study and, above all, this concerns the temperature response of CO_2_ emission activity of xylotrophic fungi to an increase in temperature. This determined the purpose of this work—an analysis of the relationships between the CO_2_ emission activity of xylotrophic fungi and temperature, testing the hypothesis of an exponential increase in CO_2_ emission with increasing temperature in the range of 10–30 °C, which is relevant for temperate latitudes.

## 2. Materials and Methods

**Figure 1:**
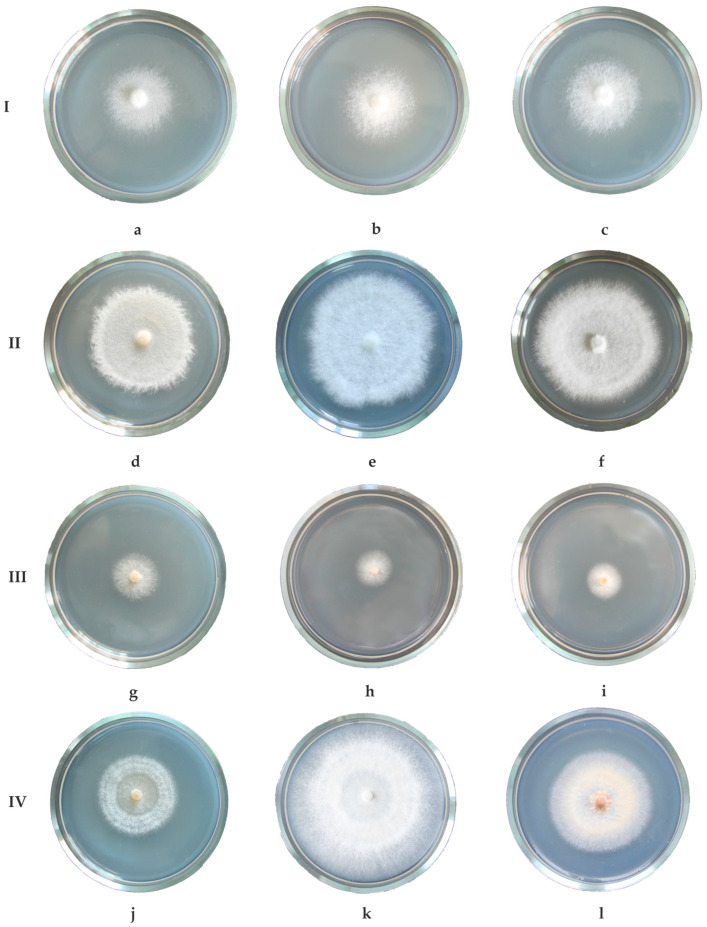
Dikaryotic mycelium of *Fomitopsis betulina* (**a**,**d**) *F. pinicola* collected on *Betula* (**b**,**e**) and *Picea* (**c**,**f**), *Phellinus igniarius* (**g**,**j**), *Fomes fomentarius* s. str. (**h**,**k**), and *Fomes inzengae* (**i**,**l**) at the beginning of the experiment (**I**,**III**) and after five days (**II**,**IV**).

A study of the temperature response of xylotrophic fungi CO_2_ emission activity was carried out on dikaryotic mycelia of *Fomes fomentarius* (L.) Fr., *F. inzengae* (Ces. and De Not.) Cooke, *Fomitopsis betulina* (Bull.) B.K. Cui, M. L. Han and Y.C. Dai, *F. pinicola* (Sw.) P. Karst., and *Phellinus igniarius* (L.) Quél. growing on wort agar (Figure 1).

Dikaryotic cultures were isolated from basidiocarps of the corresponding species of fungi using traditional methods [19] and wort (4%)–agar (2%) as a nutrient medium (MA). The use of pure cultures allows one to solve one of the most difficult problems in the study of xylotrophic fungi—the assessment of mycelium biomass in wood. When mycelium develops on artificial nutrient media, an indicator of its biomass can simply be assessed by the area it occupies in Petri dishes.

The species identification of basidiocarps was determined on anatomical and morphological characteristics [20], and their species names are given according to Index Fungorum [21]. *F. fomentarius* strains were typed using ITS region sequencing; according to phylogenetic analysis, strains collected on *Populus* L. belong to *F. inzengae* and on *Betula* L. belong to *F. fomentarius* sensu stricto—two cryptic taxa [22,23,24].

The analysis scheme was as follows. Petri dishes (9 cm in diameter, 3 for each strain) were inoculated with a piece of agar (about 5 mm) with the mycelium of the fungus being studied and kept for several days at +25 °C. When the mycelium began to grow around the inoculum, its border was marked by felt-tip pen on the underside of the Petri dishes, and dishes were placed in open exposure chambers with a volume of 0.5 L and placed in a thermostat at +10 °C for 2 h. Then, the chambers were sealed, their CO_2_ content was measured, and they were placed in a thermostat at +10 °C for one hour. At the end of the exposure, CO_2_ measurements in the chambers were made again, after which they were opened and left with closed Petri dishes inside for a day in a thermostat at +10 °C. After 24 h, the size of the mycelia in the Petri dishes was measured, the borders were marked, the chambers were closed, CO_2_ was measured, and they were placed in a thermostat at +10 °C for an hour. At the end of the exposure, the CO_2_ content in the chambers was measured again. According to the same scheme, the growth of mycelium and its emission activity were assessed at +20 °C, +25 °C, +30 °C and +35 °C.

The CO_2_ content in the chambers was measured using a Gasmet DX-4030 FTIR spectrometer (Gasmet Technologies Oy, Finland) with an accuracy of ±50 ppm. The emission activity of the mycelium was assessed by the difference in CO_2_ concentration in the chambers at the beginning of the exposure and at the end and was calculated in μg of CO_2_, taking into account the volume of the exposure chambers and Petri dishes and the exposure duration.

Specific CO_2_ emission was calculated in µg CO_2_/cm^2^/h by Equation (1):

SEA = ΔCO_2_ × (V_1_ − V_2_)/Vm × M/S × 0.27 × 273/T, (1)

The total CO_2_ emission, or the total amount of carbon dioxide emitted by the mycelium, was calculated in µg CO_2_/h by Equation (2):

TEA = ΔCO_2_ × (V_1_ − V_2_)/Vm × M × 0.27 × 273/T, (2)

where SEA is the specific CO_2_ emission, TEA is the total CO_2_ emission, ΔCO_2_ is the amount of CO_2_ released by the mycelium during exposure (ppm/h), V_1_ is the chamber volume (l), V_2_ is the sample volume (l), Vm is the molar volume (22.4 l/mol), M is the molar mass of CO_2_ (44 g/mol), S is the area occupied by mycelium (cm^2^), and T is the temperature in Kelvin (K).

The temperature coefficient (Q_10_) of specific CO_2_ emission, showing the multiplicity of its change with a temperature increase of 10 °C, was calculated by Equation (3):

Q_10_SEA = SEA_1_/SEA_2_, (3)

where Q_10_SEA is the temperature coefficient of specific emission, SEA_1_ is the specific emission at 10 and 20 °C, and SEA_2_ at 20 and 30 °C, respectively.

The temperature dependence of mycelium growth was assessed by the increase in the area it occupied on MA during the day (cm^2^/day) and by the temperature coefficient (Q_10_) of growth, calculated by the similar Equation (4):

Q_10_SM = V_2_/V_1_, (4)

where Q_10_SM is the temperature coefficient of growth, V_1_ is the intensity of mycelium growth (cm^2^/day) at 10 and 20 °C, and V_2_ is the intensity of mycelium growth at 20 and 30 °C, respectively.

Statistical data processing was performed using the Statistica 10.0 program (StatSoft Inc., Tulsa, OK, USA). Arithmetic means (m) are given with standard errors (SE). The Pearson correlation coefficient (r) was used to characterize the relationships between variables. Student’s *t*-test was used for pairwise comparisons; one-way analysis of variance (ANOVA) was used for multiple comparisons of means. The correspondence of the CO_2_ emission activity temperature dynamics with a linear (SEA = a + b × t, where t is the temperature in degrees Celsius) or exponential (TEA = a × exp(b × t)) regression model was assessed on coefficient of determination or R^2^—a statistical measure correspondence of regression line to the actual data. When describing the results of statistical evaluation, the values of the corresponding criteria and their significance are given.

## 3. Results

**Figure 2:**
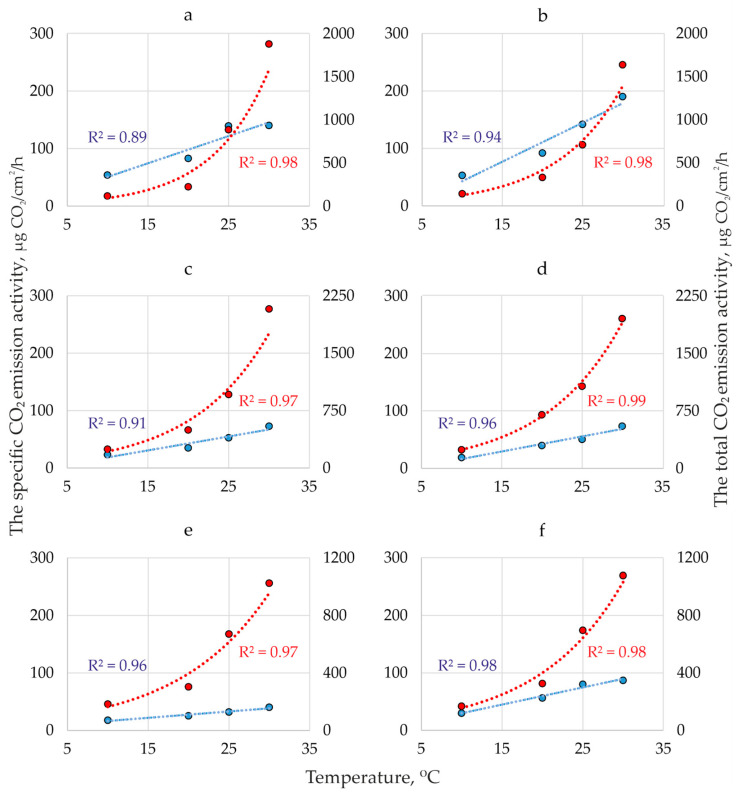
Dynamics of specific (blue) and total (red) CO_2_ emission activity (the averages based on three replicates) of dikaryotic mycelium at MA in the range of 10–30 °C of *Fomes fomentarius* sensu stricto (**a**), *F. inzengae* (**b**), *Fomitopsis pinicola* (collected on *Betula*) (**c**), *Fomitopsis pinicola* (collected on *Picea*) (**d**), *Fomitopsis betulina* (**e**), and *Phellinus nigricans* (**f**).

Figure 2 shows the dynamics of CO_2_ emission activity of five species of dikaryotic mycelium at MA in the range of 10–30 °C. It is seen that the temperature dynamics of total (TEA) and specific (SEA) CO_2_ emission activity are significantly different: SEA is linear (determination coefficient 0.91–0.98), and TEA is exponential (determination coefficient 0.94–0.99).

Table 1, Table 2 and Table 3 show that the temperature raise from 10 to 20 °C increased the specific CO_2_ emission activity of mycelium from 1.3 (*F. betulina*) to 2.1 (*F pinicola*, collected on *Picea*), on average 1.8 times. An increase in temperature from 20 to 30 °C enhances SEA by 1.2 (*F. fomentarius* s. str.)–1.9 (*F. pinicola*, collected on *Picea*) on average 1.6 times. In other words, the temperature coefficient of the specific CO_2_ emission activity of the mycelium of the studied group of xylotrophic fungi ranges from 1.6 to 1.8. An increase in temperature from 10 to 30 °C (3 times) causes a corresponding increase in SEA—2.9 times. At 35 °C, SEA decreases in some of the analyzed fungi, while in *F. betulina* and *F pinicola* (collected on *Betula*) it remains at the same level as at 30 °C.

The specific CO_2_ emission activity of the mycelium does not show any relationship connection with its size, in our case, with its area. Thus, in the *F. betulina* strain at 10 °C SEA of the same level (18–19 µg CO_2_/cm^2^/h) for mycelium with an area of 10 and 13 cm^2^, and at 20 °C, it is equal to 24–25 µg CO_2_/cm^2^/h for mycelium with an area of 13 and 21 cm^2^ (Table 1). In *F. pinicola* (strain collected on *Picea*) at 10 °C, SEA equal to 19 μg CO_2_/cm^2^/h is recorded in mycelium with an area of 11 and 14 cm^2^, as well as in the strain collected on *Betula* 23 μg CO_2_/cm^2^/h in mycelium of 13 and 18 cm^2^.

The same is observed at 30 °C: in the strain collected on *Betula*, the mycelium of 29 cm^2^ and 38 cm^2^ has SEA equal to 73 µg CO_2_/cm^2^/h, as well as in the strain collected on *Picea* 73–74 µg CO_2_/cm^2^/h in the mycelium with an area of 27 cm^2^ and 35 cm^2^ (Table 2). There is also no relationship between SEA and mycelium area in the *F. fomentarius* s. str. and *F. inzengae* strains (Table 3).

The specific CO_2_ emission activity depends on the species of fungus, and in the range of 10–20 °C, its average value varies from 27.5 ± 1.95 (*F. betulina*) to 118.8 ± 9.53 μg CO_2_/cm^2^/h (*F. inzengae*). In *F. betulina*, *F. pinicola*, it is significantly—F_(1, 142)_ = 90.160, *p* = 0.001—lower (varies from 27.5 ± 1.95 to 47.4 ± 4.19, on average 40.3 ± 2.32 μg CO_2_/cm^2^/h) than in *F. fomentarius* s. str. and *Ph. igniarius* (varies from 60.1 ± 4.59 to 118.8 ± 9.53, on average 95.4 ± 5.31 µg CO_2_/cm^2^/h). At the same time, *F. pinicola* strains isolated from basidiocarps collected on *Betula* and *Picea* have an SEA of the same level (Table 2).

The response of total CO_2_ emission activity to an increase in temperature is more pronounced than in the case of specific activity. If, with an increase in temperature from 10 to 20 °C, SEA, as noted, increases by 1.3–2.1 times on average 1.8 times only, then TEA increases by 3 (*F. betulina*)–6 (*F. fomentarius* s. str.) times, on average 4 times. When the temperature increases from 20 to 30 °C, TEA increases by 2 (*F. betulina*)–4 (*F. fomentarius* s. str., *F. pinicola*), on average 3 times, while SEA 1.6 times. An increase in temperature from 10 to 30 °C enhances the TEA of the mycelium of *F. betulina* and *Ph. igniarius* by 6, *F. pinicola* by 10, *F. inzengae* by 13, and *F. fomentarius* s. str. by 21 times—on average 11 times. At the same time, as noted above, SEA will increase by 2.9 times. Like SEA, TEA reaches its maximum at 30 °C; at 35 °C, it decreases or remains at the same level as at 30 °C. TEA varies depending on the fungus species: the highest (2000–3000 μg CO_2_/h) in the mycelium of *F. fomentarius* s. str., *F. inzengae*, *F. pinicola*, and 2–3 times lower (does not exceed 1000 μg CO_2_/h) in the mycelium *F. betulina* and *Ph. igniarius* (Table 1, Table 2 and Table 3).

The total emission activity of the mycelium depends not only on temperature but also on its size. For example, at 20 °C, an increase in the mycelium area of *F. betulina* by 1.6 times (from 13 to 21 cm^2^) is accompanied by a similar 1.6-fold increase in TEA (from 303 to 490 μg CO_2_/h). An increase in mycelium size in *Ph. igniarius* by 1.5 times (from 6 to 9 cm^2^) at 20 °C leads to a rise in its TEA by 1.6 times (Table 1). In *F. pinicola* at 20 °C, an increase in the size of the mycelium by 1.2–1.3 times is accompanied by an increase in *TEA* by 1.2–1.5 times (Table 2). An increase in TEA proportional to the increase in mycelium area is also observed in the *F. fomentarius* s. str. and *F. inzengae* strains (Table 3).

The size of the mycelium reflects the intensity of its growth, which is positively related to temperature. The correlation coefficient of the daily increase in mycelium area with temperature for *F. betulina* is 0.61, *F. pinicola* is 0.57 (strain collected on *Picea*)–0.76 (strain collected on *Betula*), *F. inzengae* and *Ph. igniarius* 0.85, and *F. fomentarius* s. str. 0.97. The temperature coefficient (Q_10_) of mycelium growth with an increase in temperature from 10 to 20 °C varies from 1.3 (*F. inzengae*) to 2.0 (*F. fomentarius* s. str.) and on average is 1.5; it has the same average value when the temperature increases from 20 to 30 °C. At 30 °C, the growth rate of *F. betulina* and *Ph. igniarius* mycelium reaches its maximum, as well as *F. pinicola*, *F. inzengae*, *F. fomentarius* s. str. at 35 °C (Table 1, Table 2 and Table 3).

## 4. Discussion

There is an opinion that for the majority of representatives of the boreal microbiota, adapted to an average summer temperature of about +15 °C, an increase in temperature to 30 °C will be tantamount to temperature shock [5]. However, as the results of this study show, in the range of 10–30 °C, xylotrophic fungi respond positively to increased temperature. Thus, in this range, the specific CO_2_ emission activity of the mycelium, which is an indicator of its respiratory activity, increases on average 1.7 times with an increase in temperature of 10 °C and 3 times with its increase from 10 to 30 °C: 1.7 × 1.7 = 2.9. In other words, the specific CO_2_ emission activity of the mycelium of xylotrophic fungi obeys the Van’t Hoff rule, and this determines the linear nature of its temperature dynamics. The specific emission activity of the mycelium does not depend on the size of the mycelium, and its relationship with temperature is described by the following Equation (5):

SEA_T2_ = SEA_T1_ × Q_10_SEA ^(T2−T1)/10^, (5)

where SEA_T1_ and SEA_T2_ are specific CO_2_ emission at temperature T_1_ and T_2_, respectively; Q_10SEA_—temperature coefficient of specific CO_2_ emission activity.

Thus, the only driver of the specific CO_2_ emission activity of the mycelium of xylotrophic fungi is temperature, or rather its temperature sensitivity, the indicator of which is the temperature coefficient. The latter, in the range of 10–30 °C, varies depending on the species from 1.2 to 2.1, with an average of 1.7. We also recorded similar Q_10_ values of specific CO_2_ emission activity when analyzing the gas exchange of wood residues destroyed by xylotrophic fungi: 2.0–2.1 [11]. The close positive relationship between specific emission activity and temperature determines its unstable nature. One of the results of this is a daily fluctuation in the intensity of CO_2_ gas exchange of wood residues: an increase in the daytime and a decrease in the night.

The total CO_2_ emission activity of the mycelium is determined by its specific emission activity and size. Accordingly, its temperature dynamics have two drivers: the temperature sensitivity of (a) specific emission and (b) mycelium growth, indicators of which are the temperature coefficients of specific emission and mycelium growth. The relationship between total emission and temperature is described by the following Equation (6):

TEA_T2_ = TEA_T1_ × Q_10_SEA ^(T2−T1)/10^ × Q_10_SM ^(T2−T1)/10^, (6)

where TEA_T2_ and TEA_T1_ are the total CO_2_ emission at temperature T_1_ and T_2_, respectively; Q_10_SEA and Q_10_SM are the temperature coefficient of specific CO_2_ emission and mycelium growth, respectively.

Depending on the species of fungi, the Q_10_ of mycelium growth ranges from 1.3 to 2.0. On average, it is 1.5 and almost identical to Q_10_ of specific emission: 1.7. Therefore, an increase in temperature in the range of 10–30 °C causes an almost equal increase in two unidirectional processes—specific CO_2_ emission activity and mycelium growth. Their joint action causes an exponential increase in total CO_2_ emissions. The dependence on the mycelium size determines another very important feature of the total CO_2_ emission activity of xylotrophic fungi. Because the growth of mycelium means an irreversible increase in its size and mass, the temperature dynamics of the total emission also have the character of a directed, irreversible process. The total emission reaches its maximum at 30–35 °C, a temperature at which both the maximum specific CO_2_ emission activity and the size of the mycelium are observed.

## 5. Conclusions

In the range of summer temperatures (10–30 °C) that are relevant for temperate latitudes, the CO_2_ emission activity of xylotrophic fungi is closely and positively related to temperature. Their specific CO_2_ emission activity is determined by the respiratory activity of the mycelium and does not depend on its size. The only driver of specific emissions is temperature, an increase in which causes its proportional (linear) increase. The total CO_2_ emission activity, which is an indicator of the amount of CO_2_ emitted, depends on the size and specific emission activity of the mycelium. It has the character of an irreversible, directional process that increases exponentially with increasing temperature to 30–35 °C. This gives fairly strong grounds to assume that climate warming will lead to an exponential increase in the CO_2_ emission activity of woody debris, which, in turn, could potentially contribute to the acceleration of climate change.

## Figures and Tables

**Table 1:** The total and specific CO_2_ emission activity of the dikaryotic mycelium of *Fomitopsis betulina* and *Phellinus igniarius* and its relationship with their area and temperature.

Temperature, °C	Time	*Fomitopsis betulina*		Specific Emission, µg CO_2_/cm^2^/h	*Phellinus igniarius*		Specific Emission, µg CO_2_/cm^2^/h
		Mycelium Area, cm^2^	Total Emission, µg CO_2_/h		Mycelium Area, cm^2^	Total Emission, µg CO_2_/h	
10	0 h	10 ± 0.5	182 ± 5.0	18 ± 0.8	6 ± 0.2	168 ± 12.0	30 ± 2.8
	24 h	13 ± 1.4	233 ± 1.1	19 ± 2.0	6 ± 0.2	165 ± 47.1	30 ± 8.7
20	0 h	13 ± 1.4	303 ± 69.2	25 ± 7.1	6 ± 0.2	326 ± 21.9	56 ± 4.3
	24 h	21 ± 1.6	490 ± 10.2	24 ± 2.2	9 ± 0.5	517 ± 11.2	60 ± 4.9
25	0 h	21 ± 1.6	671 ± 52.5	32 ± 2.6	9 ± 0.5	697 ± 20.1	80 ± 2.7
	24 h	27 ± 3.0	789 ± 34.7	30 ± 2.6	13 ± 0.8	988 ± 46.8	80 ± 5.2
30	0 h	27 ± 3.0	1024 ± 56.4	40 ± 6.0	13 ± 0.8	1077 ± 16.9	87 ± 7.3
	24 h	35 ± 3.9	1076 ± 87.3	33 ± 5.9	18 ± 0.6	1062 ± 49.6	60 ± 4.7
35	0 h	35 ± 3.9	1049 ± 129.5	32 ± 6.8	18 ± 0.6	946 ± 92.8	53 ± 4.2
	24 h	35 ± 3.1	917 ± 27.4	27 ± 2.4	19 ± 0.5	645 ± 24.7	35 ± 0.4

**Table 2:** The total and specific CO_2_ emission activity of the dikaryotic mycelium of *Fomitopsis pinicola* collected on *Betula and Picea* and its relationship with their area and temperature.

Temperature, °C	Time	*Fomitopsis pinicola* (*Betula*)		Specific Emission, µg CO_2_/cm^2^/h	*Fomitopsis pinicola* (*Picea*)		Specific Emission, µg CO_2_/cm^2^/h
		Mycelium Area, cm^2^	Total Emission, µg CO_2_/h		Mycelium Area, cm^2^	Total Emission, µg CO_2_/h	
10	0 h	11 ± 1.3	244 ± 34.7	23 ± 6.0	13 ± 0.8	242 ± 17.6	19 ± 0.5
	24 h	14 ± 1.3	319 ± 20.4	23 ± 3.7	18 ± 1.2	336 ± 22.4	19 ± 2.5
20	0 h	14 ± 1.3	495 ± 22.8	35 ± 1.7	18 ± 1.2	699 ± 44.7	40 ± 2.9
	24 h	18 ± 1.8	740 ± 36.9	41 ± 4.3	21 ± 1.4	856 ± 0.6	40 ± 2.5
25	0 h	18 ± 1.8	959 ± 62.3	52 ± 2.2	21 ± 1.4	1072 ± 18.5	50 ± 2.7
	24 h	29 ± 2.3	1698 ± 22.0	59 ± 4.4	27 ± 1.3	1416 ± 26.6	53 ± 2.3
30	0 h	29 ± 2.3	2076 ± 13.1	73 ± 6.4	27 ± 1.3	1955 ± 37.7	73 ± 2.6
	24 h	38 ± 2.5	2736 ± 44.3	73 ± 6.5	35 ± 1.4	2551 ± 35.0	74 ± 3.5
35	0 h	38 ± 2.5	2771 ± 34.1	74 ± 5.7	35 ± 1.4	2382 ± 49.2	69 ± 1.6
	24 h	42 ± 2.3	2670 ± 83.9	65 ± 5.2	43 ± 0.9	2290 ± 33.7	53 ± 1.2

**Table 3:** The total and specific CO_2_ emission activity of the dikaryotic mycelium of *Fomes fomentarius* sensu stricto and *Fomes inzengae* and its relationship with their area and temperature.

Temperature, °C	Time	*Fomes fomentarius*		Specific Emission, µg CO_2_/cm^2^/h	*Fomes inzengae*		Specific Emission, µg CO_2_/cm^2^/h
		Mycelium Area, cm^2^	Total Emission, µg CO_2_/h		Mycelium Area, cm^2^	Total Emission, µg CO_2_/h	
10	0 h	2 ± 0.1	116 ± 1.1	54 ± 4.7	3 ± 0.2	141 ± 15.2	53 ± 7.8
	24 h	3 ± 0.1	147 ± 17.5	55 ± 7.8	4 ± 0.5	250 ± 42.4	69 ± 3.6
20	0 h	3 ± 0.1	222 ± 36.3	83 ± 11.9	4 ± 0.5	328 ± 75.4	92 ± 15.2
	24 h	6 ± 0.2	681 ± 98.0	106 ± 11.8	5 ± 0.5	516 ± 13.7	104 ± 7.5
25	0 h	6 ± 0.2	884 ± 78.6	139 ± 12.0	5 ± 0.5	707 ± 53.2	142 ± 7.4
	24 h	13 ± 0.2	2064 ± 133.9	154 ± 9.2	9 ± 0.7	1269 ± 98.9	146 ± 2.7
30	0 h	13 ± 0.2	1878 ± 36.9	140 ± 3.0	9 ± 0.7	1637 ± 56.9	190 ± 13.4
	24 h	19 ± 0.8	2401 ± 41.0	127 ± 3.0	12 ± 0.8	1868 ± 26.2	155 ± 8.2
35	0 h	19 ± 0.8	2588 ± 127.5	137 ± 1.3	12 ± 0.8	1777 ± 82.6	147 ± 6.6
	24 h	25 ± 1.3	2042 ± 183.5	84 ± 10.7	16 ± 1.0	1650 ± 78.9	102 ± 10.5

## Data Availability

Data are contained within the article.
